# Clinical Assessment of Judgment in Adults and the Elderly: Development and Validation of the Three Domains of Judgment Test—Clinical Version (3DJT-CV)

**DOI:** 10.3390/jcm12113740

**Published:** 2023-05-29

**Authors:** Simon-Pierre Bernard-Arevalo, Robert Jr Laforce, Olivier Khayat, Vital Bouchard, Marie-Andrée Bruneau, Sarah Brunelle, Stéphanie Caron, Laury Chamelian, Marise Chénard, Jean-François Côté, Gabrielle Crépeau-Gendron, Marie-Claire Doré, Marie-Pierre Fortin, Nadine Gagnon, Pierre R. Gagnon, Chloé Giroux, Léonie Jean, Geneviève Létourneau, Émilie Marceau, Vincent Moreau, Michèle Morin, Christine Ouellet, Stéphane Poulin, Steve Radermaker, Katerine Rousseau, Catherine Touchette, Alexandre Dumais

**Affiliations:** 1 Research Center of the Institut Universitaire en Santé Mentale de Montréal, Montreal, QC H1N 3V2, Canada; simon-pierre.bernard.1@ulaval.ca (S.-P.B.-A.);; 2 Faculty of Medicine, Université Laval, Quebec City, QC G1V 0A6, Canada; 3 Department of Neurological Sciences, CHU de Québec—Université Laval, Quebec City, QC G1J 1Z4, Canada; 4 Faculty of Medicine, Université de Montréal, Montreal, QC H3C 3J7, Canada; 5 Centre Intégré Universitaire de Santé et de Services Sociaux de la Capitale-Nationale, Quebec City, QC G1C 3S2, Canada; 6 Research Center of the Institut Universitaire de Gériatrie de Montréal, Montreal, QC H3W 1W4, Canada; 7 Centre Intégré Universitaire de Santé et de Services Sociaux du Nord-de-l’Île-de-Montréal, Montreal, QC H3L 1K5, Canada; 8 Department of Psychiatry, Centre Hospitalier Universitaire de Montréal, Montreal, QC H2X 0C1, Canada; 9 Centre Intégré Universitaire de Santé et de Services Sociaux de l’Est-de-l’Île-de-Montréal, Montreal, QC H1T 2M4, Canada; 10 Institut de Réadaptation en Déficience Physique de Québec, Quebec City, QC G1W 1P7, Canada; 11 Centre Intégré de Santé et de Services Sociaux de Chaudière-Appalaches, Sainte-Marie, QC G6E 3E2, Canada; 12 Institut National de Psychiatrie Légale Philippe-Pinel, Montreal, QC H1C 1H1, Canada

**Keywords:** cognitive impairment, cognitive neuroscience, judgment assessment, neuropsychiatric assessment, participatory research, telemedicine and neuropsychological tests

## Abstract

(1) Background: This article discusses the first two phases of development and validation of the Three Domains of Judgment Test (3DJT). This computer-based tool, co-constructed with users and capable of being administered remotely, aims to assess the three main domains of judgment (practical, moral, and social) and learn from the psychometric weaknesses of tests currently used in clinical practice. (2) Method: First, we presented the 3DJT to experts in cognition, who evaluated the tool as a whole as well as the content validity, relevance, and acceptability of 72 scenarios. Second, an improved version was administered to 70 subjects without cognitive impairment to select scenarios with the best psychometric properties in order to build a future clinically short version of the test. (3) Results: Fifty-six scenarios were retained following expert evaluation. Results support the idea that the improved version has good internal consistency, and the concurrent validity primer shows that 3DJT is a good measure of judgment. Furthermore, the improved version was found to have a significant number of scenarios with good psychometric properties to prepare a clinical version of the test. (4) Conclusion: The 3DJT is an interesting alternative tool for assessing judgment. However, more studies are needed for its implementation in a clinical context.

## 1. Introduction

Judgment is a cognitive skill that people use on a daily basis. It allows people to assess a situation within its context and identify possible solutions in order to make a decision. Judgment ensures the individual and social functioning of people and protects them from existing dangers [1]. Consequently, a judgment capacity altered by a pathology may result in significant limitations in a person’s instrumental activities of daily living, thus compromising their autonomy, their ability to remain at home, and their overall safety [2].

Three domains of judgment are frequently identified in the literature: practical, moral, and social. In general, authors recognize these as different constructs (for example, see [3,4,5,6,7,8,9,10,11,12,13]). Anatomical [6,14,15,16,17,18,19], developmental [3,4,5,9,20,21,22], and pathological [23,24,25,26,27] bases support this functional distinction (see Table 1). Moral judgment is involved in situations where the individual will have to assess the appropriateness of a behavior relative to the ideas conveyed by society about right and wrong [7]. Social judgment will be called into action in conditions where the subject will have to determine whether a behavior respects the non-moral standards underlying interactions between people, such as rules of politeness or decorum [10]. Finally, practical judgment will be applied in practical situations in an individual’s daily life and involve a decision that does not have a socio-moral connotation in the foreground [13]. On the other hand, judgment must be distinguished from decision-making [23,28]. The former concerns the assessment of various solutions that have been generated in the face of a given situation [23]. Decision-making concerns the individual’s choice of one of these options [23]. Although these two operations are part of a continuum, their distinction is relevant because some individuals can make bad decisions while maintaining their judgment abilities.

**Table 1:** Neuroanatomical, developmental, and pathological bases support the argument that the three main domains of judgment are different constructs.

	Practical Judgment	Moral Judgment	Social Judgment
Neuroanatomical regions involved	Prefrontal cortex, parietal cortex, thalamus, and striatum (ventral and dorsal) [14,15,16].	Dorsomedial prefrontal cortex, ventromedial prefrontal cortex, temporoparietal junctions, left amygdala, precuneus, and left lateral orbitofrontal cortex to its extension into the region adjacent to the interior insula [17].	Medial prefrontal cortex, lateral part of the left orbitofrontal cortex, left amygdala, temporoparietal junction, middle and superior temporal gyrus, posterior cingulate cortex, lingual gyrus, and fusiform gyrus [6,18,19].
Developmental aspects	Effect of age and socio-economic status on danger perception in children. 5–6-years-old-children identify and recognize most of the dangers encountered in daily life. The same is not true for 3–4-years-old-children [20,21,22].	From the age of 34 months, children would be able to distinguish transgressions of moral rules from violations of social rules based on the criterion that a breach of a moral rule is transgressed in several contexts and not only in the one proposed in the evaluation tests [5].	At the age of 42 months, children’s ability to distinguish between a transgression of a moral norm and a violation of a social rule would increase, particularly in relation to lesser seriousness attributed to a transgression of a social norm and that this violation would be legitimate in the presence of a rule or an authority figure allowing it [5].
Pathological aspects	Could be affected in mild Alzheimer’s disease (amnestic variant) [23].	Would be affected in even mild frontotemporal dementia due to social-cognitive deficits [24,25,26].	Would be affected in frontotemporal dementia, but more markedly in subjects with behavioral variant [27].

**Figure 1:**
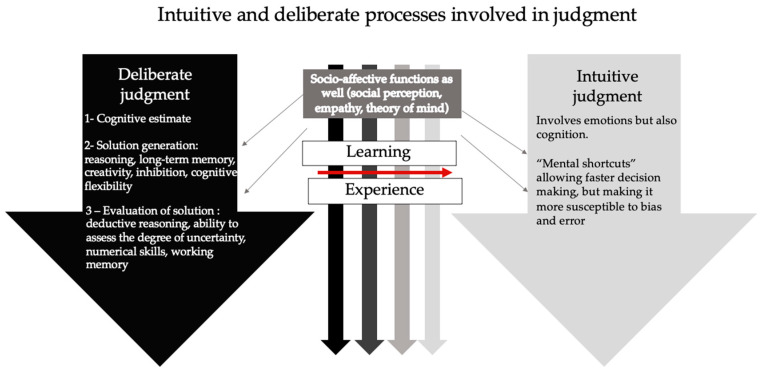
Mental operations and processes involved in judgment. Judgment involves two types of processes: deliberate (left arrow) and intuitive (right arrow). Intuitive processes allow instantaneous decision-making in simple or familiar contexts. Deliberate processes, for their part, enable thoughtful and creative decision-making in new or complex contexts. They are made of three steps: cognitive estimation, generation of solutions and evaluation of solutions. Socio-affective functions (such as emotion recognition, affective regulation, empathy, or theory of mind) are involved in both deliberate and intuitive judgments. Learning and experience allow a switch from using deliberate processes to intuitive processes to assess a situation.

Cognitively, judgment involves two types of processes: intuitive and deliberate [29,30,31,32,33] (see Table 2 and Figure 1). The first allows one to quickly evaluate a situation and the possible solutions in order to make an instantaneous decision [29,30,31,32,33]. Intuitive processes often come into play in simple, familiar contexts or situations for which the person has received specific training [33]. Deliberate processes, for their part, enable thoughtful and creative decision-making in new or complex contexts [23]. Deliberate processes are made up of three steps: cognitive estimation, generation of solutions, and assessment of solutions [23,34,35] (see Figure 1). The first step, cognitive estimation, determines whether a situation will generate a problem and need special attention [36]. The second stage, the generation of solutions, will require reasoning skills [37], long-term memory integrity [35], good creativity [35], and inhibition [38]. The third step, assessment of solutions, will involve weighing the advantages and disadvantages of each of the possible options to solve the situation. To this end, deductive reasoning skills [35,38,39] as well as the ability to assess the degree of uncertainty of these advantages and disadvantages will be required [36,40]. Numerical skills could also be of use depending on the situation [41,42]. Furthermore, working memory will be involved in the generation of solutions as well as in their assessment [43]. As for socio-cognitive processes such as emotion recognition, affective regulation, empathy, or theory of mind, they will be more needed in tasks involving an individual’s moral or social judgment [10,11,25].

Despite the importance of the three main domains of judgment, few validated tools have been developed to assess them in clinical practice. The only ones we have identified were designed to measure practical judgment and include the Neurobehavioral Cognitive Status Exam (NCSE-JQ) [44,45], the Problem Solving Subtest of the Independent Living Scale (ILS-PS) [46], the Judgment/Daily Living Test of the Neuropsychological Assessment Battery (NAB-JDG) [47], the Test of Practical Judgment (TOP-J) [38], the Kitchen Picture Test (KPT) [48], the Verbal Test of Practical Judgment (VPJ) [2], and the Judgment Assessment Tool (JAT) [23]. These tests are made up of scenarios presenting situations to which the individual might be exposed in their daily life, and which require decision-making that considers case background. However, several of these tools have psychometric weaknesses (see Table 3). For example, the NCSE-JQ, NAB-JDG, and VPJ have low internal consistency [2,23,45]. Furthermore, the NCSE-JQ, NAB-JDG, and ILS-PS have content validity problems these tests assess general knowledge more than judgment [23]. It should be noted that two tests have good psychometric properties, specifically the TOP-J and the JAT (see Table 3). However, these two instruments do not assess moral or social judgment. Moreover, they are not adapted to digital technologies and cannot be administered remotely, which has become particularly important following the COVID-19 pandemic (in this sense, see [49]). Finally, these tests may lack relevance or may not be acceptable to certain groups because they have not been co-constructed in collaboration with patients or their relatives or with various clinicians with expertise in the field.

The objective of the present study was to develop and validate an instrument in French that can be used to measure the three main domains of judgment (practical, moral, and social). We have named this tool the Three Domains of Judgment Test (3DJT) (in French: *Test d’évaluation des trois domaines du jugement* or *TÉ3J*). As a whole, the 3DJT aims to improve on the weaknesses of instruments developed to date by having good content validity, internal consistency, ecological validity, and acceptability for eventual administration to patients. It is a computerized instrument with the potential to be administered remotely and can eventually be adapted for functional neuroimaging studies. Finally, it is a tool that has been co-constructed with expert clinicians from a variety of disciplines and backgrounds, as well as non-cognitively impaired individuals, and that eventually intends to incorporate the participation of patients and other stakeholders in its improvement. This paper will discuss the first two phases of the development and validation of a potential short 3DJT version that could be used in clinical trials (3DJT Clinical Version, or 3DJT-CV). In Phase 1, we presented the 3DJT to experts in adult and elderly cognition for an initial assessment. An improved version of the tool was then developed and used for Phase 2, which consisted of a validation study with 70 subjects without cognitive impairment.

At this stage, our hypothesis was that the items in 3DJT would measure each of the three major domains of judgment (practical, moral, and social) with good content validity and acceptability for eventual administration to patients. We also hypothesized that 3DJT would show good internal consistency and that correlations between tool items and cognitive tests used for the concurrent validity primer would indicate that 3DJT could be a good measure of judgment. Finally, we hypothesized that a sufficient number of items would have sufficiently good psychometric properties to consider a short version of the test that can be used in clinical settings (3DJT-CV).

## 2. Materials and Methods

### 2.1. Participants

#### 2.1.1. Participants in Phase 1 (Experts’ Evaluation)

A group of 32 experts were recruited from 13 different professional settings located in four cities in the province of Quebec, Canada. These experts had cognitive neurology, neuropsychiatry, geriatrics, geriatric psychiatry, consultation-liaison psychiatry, neuropsychology, or occupational therapy as their areas of practice. The study was conducted by the *Centre de recherche de l’Institut universitaire en santé mentale de Montréal*. All participants provided written informed consent as approved by the Research Ethics Board of the *Centre intégré universitaire de santé et de services sociaux de l’Est-de-l’Île-de-Montréal* (project number assigned by CEMTL REB: 2021-2581).

#### 2.1.2. Participants in Phase 2 (Psychometric Evaluation of the Items)

Subjects were recruited from the community through online advertisements, social networks, and word of mouth. Subjects had to meet the following inclusion criteria: (1) at least 18 years old; (2) French-speaking or comfortable with the French language; (3) no cognitive impairment as defined by the MoCA (Montreal Cognitive Assessment [50]) score of 26 or higher; (4) no past neurological or psychiatric history. Moreover, subjects had to refrain from using any recreational drugs for at least 72 h before assessment and alcohol for 24 h before assessment. If a subject had a MoCA lower than 26, a team discussion was held regarding inclusion in the study based on the results obtained in the other cognitive assessments conducted. The study was conducted at the Centre de recherche de l’Institut universitaire en santé mentale de Montréal. All participants provided written informed consent as approved by the Research Ethics Board of the Centre intégré universitaire de santé et de services sociaux de l’Est-de-l’Île-de-Montréal (project number assigned by CEMTL REB: 2021-2581).

### 2.2. Materials

#### 2.2.1. Materials for Phase 1 (Experts’ Evaluation)

*Original Version of the Three Domains of Judgment Test (3DJT)*: The original version of the 3DJT consisted of 72 items divided into three subtests, each containing 24 items, with each subtest measuring one of the three domains of judgment (practical, moral, and social). The items were presented in French in a computerized format and could be viewed on a 2D computer screen. Prior to the test, subjects were given written and audio instructions, after which items were presented. Each item contained four screens. On the first screen, a situation was presented through images, and a written statement was also read to the participant using audio. On the second screen, a solution to the situation was offered, and the participant was asked to decide whether or not he or she would choose this solution given the situation (a dichotomous or type D question). A maximum of six seconds were allowed to answer the question. A third screen asked the subject to justify the answer given to the previous question within a maximum of one minute (justification or type J question). Finally, a fourth and last screen could ask the subject either to generate as many solutions as possible to the situation (type G question) or to reassess their answer after changing a contextual item in the initial item scenario (type A question). For type G and type A questions, the subject had a maximum of one minute to give their answer. Time limits for answering questions were established to better structure the evaluation while minimizing the possible effects of social desirability. In developing the test, efforts were made to make items as close as possible to everyday life situations and to place more emphasis on the processes underlying the judgment than on decision-making itself. In addition, each subtest (practical, moral, and social) contained the same number of items as a G and A question (12 items for each question type).

*Expert Assessment Sheet:* A questionnaire was prepared to allow experts to assess test items and provide general comments about the test. For each item, the expert was asked to determine whether it was a situation that assessed practical judgment, moral judgment, social judgment, more than one domain of judgment, or whether the item did not assess judgment. The expert was also asked to assess the relevance, acceptability, ecological validity, clarity, and difficulty of the item, as well as the relevance and quality of the images used to illustrate it. To do this, a Likert scale ranging from 0 to 5 was used (see Figure S1 in Supplementary Materials). Finally, the expert could give general comments on the item. A final section of the questionnaire allowed experts to address the format and test instructions and give their overall comments on the tool.

#### 2.2.2. Materials for Phase 2 (Psychometric Evaluation of Items)

*Modified Version of 3DJT*: An improved version of the 3DJT developed from expert feedback and suggestions was used. These include comments made in the expert assessment sheets as well as during the focus groups (as further described in Section 3.1). The improved version included 56 enhanced items accepted by the experts. Additionally, type A scenarios were replaced with type E (evaluation of consequences) items. These items measure individuals’ abilities to assess the advantages and disadvantages of solutions. Finally, in the new version of the 3DJT, we increased the allocated time to answer type D questions to 8 s. Table 4 provides three sample items, each assessing one of the three main domains of judgment (practical, moral, and social).

The 56 enhanced items were randomly separated into two versions (A and B) of 28 scenarios, each divided into four blocks of seven items to be administered to participants. Each participant completes one of the two versions, i.e., 28 items. We decided to separate the scenarios into two versions for reasons of feasibility and acceptability to the participants (tolerability and cognitive fatigability). After each scenario, participants were asked to rate the quality of the item on a Likert scale ranging from 0 to 5, based on four criteria: statement clarity, ecological validity, difficulty in solving the item, and emotions triggered.

A grid was developed to rate type J (justification) questions using the following criteria:

- Content consistency of the reasoning with the answer given to the type D question;
- Understanding of the situation and the issues associated with it;
- Understanding of the situation and adaptation of the answer to the circumstances;
- Consideration of the alternatives and consequences of the chosen solution;
- Organization of thought and reasoning.

For type G questions (generation of solutions) and type E questions (evaluation of consequences), points were given for each correct answer provided by the participant.

In addition, two practice items were developed and used to explain the test to participants.

*Baseline measures:* In addition to the MoCA, a 13-item Marlowe-Crowne Social Desirability Scale version as developed by Reynolds [51,52] and a sociodemographic questionnaire were administered to participants. The Marlowe–Crowne Scale was used to control the effect of social desirability on subject answers, especially for moral judgment scenarios.

*Cognitive tests:* A concurrent validity primer was conducted with the following cognitive tests: Judgment Assessment Tool (JAT) [23], 15-Word Test (Rey) [53], Semantic Fluency Test [54], and Facial Emotions Stimuli (Ekman and Friesen) [55]. We chose the JAT over the TOP-J because of the psychometric properties of the JAT (multidimensional assessment of judgment, better internal consistency, very good inter-rater reliability), in addition to the fact that it was validated in French in a Quebec population.

*3DJT Assessment Questionnaire*: Finally, a questionnaire was administered to participants in order to obtain their comments and suggestions regarding various aspects of the modified version of the 3DJT. The results of this questionnaire will be discussed in a future article because we planned to analyze this data using a different theoretical framework and methods than those used in this paper.

### 2.3. Procedure

#### 2.3.1. Procedure of Phase 1 (Experts’ Evaluation)

A virtual meeting was held with experts to explain the various aspects of the project and their participation in the study. Following this meeting, the 32 experts were grouped into four teams of eight participants each. Members of each team were asked to assess 18 items using the assessment form described above. We decided to divide the item evaluation into four expert groups for feasibility reasons (workload for experts and facilitation of the conduct of the focus groups that were organized to discuss assessment results). After the assessment sheets were received, the results were compiled in order to prepare the discussion for the focus groups. Items were also classified as “accepted,” “eliminated,” or “borderline” according to criteria related to their content validity, relevance, and acceptability for possible administration to patients (see Table 5).

Focus groups were organized for each of the four teams to discuss the assessment results. These discussions were conducted by videoconference and recorded, and they allowed for further comments and suggestions for improving items and the test overall.

#### 2.3.2. Procedure of Phase 2 (Psychometric Evaluation of Items)

The assessment of the revised 3DJT was conducted in two meetings. At the first meeting, we collected socio-demographic information and conducted a cognitive assessment of the participants using the various tests described above. During the second meeting, one of the two improved versions of the 3DJT (A or B) was administered to subjects. For this purpose, one of the two versions was randomly assigned, balancing the age and gender of participants between groups. At the end of the meeting, subjects were asked to complete the 3DJT assessment questionnaire. For logistical reasons, the order of the meetings had to be reversed for some participants.

### 2.4. Analyses

#### 2.4.1. Analyses for Phase 1 (Experts’ Evaluation)

The results were compiled, and averages and standard deviations were calculated from the various ratings made by the experts.

#### 2.4.2. Analyses for Phase 2 (Psychometric Evaluation of Items)

Averages and standard deviations were calculated from the results of the questionnaires and cognitive tests administered. The same is true for 3DJT scores. Correlations were calculated between the scores on various 3DJT items and the scores of the various cognitive tests used in the concurrent validation primer. To determine internal consistency, a Cronbach’s coefficient was calculated for each of the subtests from the scores obtained for their constituent items. Statistical analyses were conducted using SPSS 28 software (IBM, Armonk, NY, United States). The alpha threshold was 0.05.

## 3. Results

### 3.1. Results of Phase 1 (Experts’ Evaluation)

The composition of the expert panel is presented in Table S1 of the Supplementary Materials. Of the 72 items that made up the initial version of the 3DJT, 56 were classified as “accepted” (78%), 10 as “borderline” (14%), and 6 as “eliminated” (8%). Of the “accepted” items, 18 assessed practical judgment, 18 assessed moral judgment, and 20 assessed social judgment (see Table S2 in Supplementary Materials). In addition, experts made several comments and suggestions about individual items and the test overall that were compiled and integrated to make a new and improved version of the 3DJT with the 56 “accepted” items. These comments are summarized in Table 6. Among them was the suggestion to develop a test with 6 or 12 items for use in clinic practice and to increase the time allowed for answering type D questions, which we did by increasing it to 8 s. The experts valued the fact that the 3DJT is more interested in assessing the underlying judgment processes than the actual decisions made by participants. In addition, suggestions were made to improve the ecological validity of some of the items and to consider sociocultural and generational differences. While good ecological validity of the item is, in general, desirable, it was noted that it might still be useful to have some items that are less likely to occur in everyday life but that raise important issues (e.g., important ethical or societal issues). It was also noted that it would be desirable to have items with a certain variation in difficulty levels, in particular to avoid a ceiling effect. Finally, and following discussions during the focus groups, we decided to change type A questions for questions asking participants to evaluate the positive and negative consequences of solutions (type E for “evaluation of consequences” questions). This choice was made in order to have a more quantitative measure of the judgment dimension associated with the assessment of the advantages and disadvantages of solutions and to make the question more structured to be better adapted to a clientele with cognitive impairment.

### 3.2. Results of Phase 2 (Psychometric Evaluation of Items)

**Figure 2:**
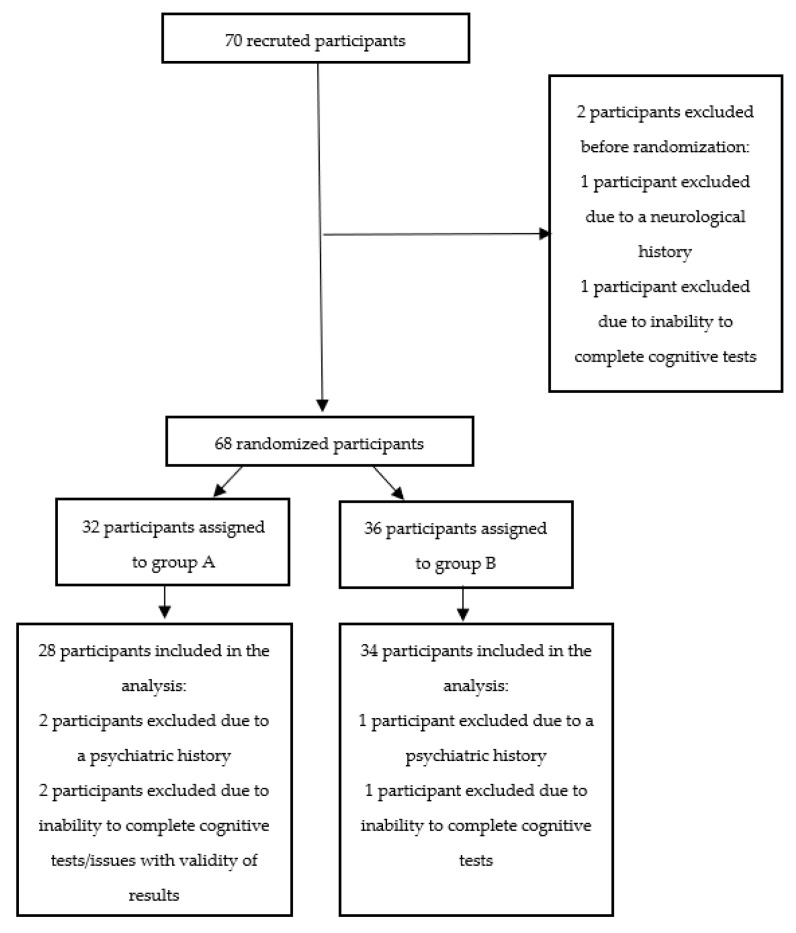
Diagram illustrating the flowchart of participants throughout the study.

Seventy subjects without cognitive impairment were recruited (see Figure 2). Sixty-two participants were included in the analysis. A total of 8 subjects were excluded due to a neurological history (*n* = 1), a psychiatric history (*n* = 3), failure to complete cognitive tests, or issues with the validity of results (*n* = 4). The two groups that took each version of the test were comparable in terms of the various socio-demographic characteristics measured in the study (see Table 7). Participants had a mean age of 39.00 years in group A and 40.74 years in group B. For each of the test versions, there was no correlation with years of study or with the score on the 13-item Marlowe–Crowne Social Desirability Scale. A negative correlation was found between the total 3DJT score and age, but only for version A of the test. Regarding the various item quality measures, the vast majority of items (48/56) scored 4/5 or higher for clarity (see Table S3 in Supplementary Materials) and 3/5 or higher (45/56) for ecological validity. Participants found items to be generally easy to medium in difficulty and low in emotion triggered.

Positive correlations with a medium effect size could be established between the 3DJT score per item and the JAT score for 22 items (39.3%) (see Table S4 in Supplementary Materials). In addition, statistical trends (*p*-values between 0.05 and 0.1) were obtained for seven more items. Furthermore, positive correlations with a medium effect size could be established between the 3DJT score per item and the semantic fluency test score for 23 items, and statistical trends were obtained for eight other items. It should be noted that there are a significant number of items for which the 3DJT score correlates positively with both the JAT score and the semantic fluency test score. Furthermore, no correlation that went against what was expected was found either with Rey’s test (number of trials needed to correctly recall the 15-word list) or with the Facial Emotions Stimuli.

Finally, internal consistency, as assessed by Cronbach’s alpha, was 0.830 and 0.841 for the practical judgment subtest (for version A and version B of the 3DJT, respectively), 0.883 and 0.776 for the moral judgment subtest, and 0.858 and 0.876 for the social judgment subtest (see Table S5 in Supplementary Materials).

## 4. Discussion

The objective of this article was to present the first two steps in the development and validation of a new tool to assess the three major domains of judgment: practical, moral, and social. A particular interest was expressed in having a test developed and validated for the French-speaking Quebec population. This process included input from 32 cognitive clinicians from a variety of disciplines and practice settings, as well as comments and suggestions from healthy participants who had used the test. To our knowledge, this is the first cognitive test to use such a broad approach. It is part of a growing trend in the health sector to develop instruments adapted to the needs of the target clientele by integrating the points of view of stakeholders [56,57]. This approach allowed us to enrich the discussions from a variety of viewpoints and enhance the final product. Furthermore, it is a tool that focuses more on assessing the integrity of the judgment processes leading to the decision than on the decision itself. By recognizing the possibility that a situation may have several possible solutions as long as these solutions adequately take into account the context and are well justified, the 3DJT is innovative by taking an approach that recognizes the pluralism that exists within society. This is especially important because of the cultural and intergenerational differences that exist in society. Finally, the 3DJT is a computerized tool that has the potential to be administered remotely, allowing, for example, access to a sub-specialized assessment in regional settings.

The first phase of our study confirmed the relevance and clinical interest of developing a test to assess the three main domains of judgment. A large number of items from the initial 3DJT version were accepted by experts because of their good content validity, relevance, and acceptability, thus corroborating the first part of our hypothesis. Furthermore, item classification by experts regarding the judgment domain assessed by the scenarios supports the argument made in the literature that practical, moral, and social judgment are three constructs that are sufficiently distinct from each other [3,4,5,6,7,8,9,10,11,12,13]. From the discussions we had during focus groups, we noted the importance of a test focused more on the assessment of underlying judgment processes than on the final result, i.e., the decision made by the participant. Finally, the co-construction of the test in collaboration with experts from various disciplines and working with diverse clienteles allowed us to broaden the discussions and bring new perspectives, particularly with regard to the consideration of cultural and intergenerational differences in the 3DJT.

The results of the psychometric study support the second part of our hypothesis that the 3DJT has good internal consistency and that the concurrent validity primer shows a tendency for it to be a good measure of judgment. However, while these results are promising, more studies are needed to confirm this latter claim. Moreover, a study among subjects with cognitive impairment will be required to confirm if our new test can be a reliable and valid tool for measuring judgment. Furthermore, in the psychometric study, we obtained medium-effect size correlations for a significant number of items between the 3DJT score and the JAT score. Such a result shows that the 3DJT and the JAT measure the same cognitive skill but with their own specificities. For example, the 3DJT assesses two additional domains of judgment (moral and social) and takes a quantitative and qualitative approach that emphasizes both the reasoning behind decision-making and the ability to generate or assess solutions. This distinguishes it from the JAT [23] and the TOP-J [38], which only assess practical judgment using a more quantitative approach. Furthermore, a significant number of the 3DJT items have a good correlation with scores on the semantic fluency test. Such a result could be explained because both tasks involve option generation [16,23]. Regarding the lack of correlation between the scores on the item-based 3DJT and those on the Rey test (number of trials needed to correctly recall the 15-word list), it should be noted that a similar result was obtained when the JAT was validated with another delayed recall measure. As for the absence of a significant correlation with the emotion recognition test, a possible explanation could be due to the 3DJT format, which would make this social-cognitive skill less necessary to solve items. The internal consistency of 3DJT subtests (Cronbach’s alpha between 0.776 and 0.883) was promising and superior to other judgment tests such as NCSE-JQ (0.04 and 0.46) [45] or NAB-JDG (0.45) [47]. Additionally, the results of Phase 2 support the last part of our hypothesis, i.e., that it is possible to identify a sufficient number of items (6 to 12 according to the experts’ request) with good psychometric properties to develop a clinical short version of the test (3DJT-CV). A short version is particularly important in light of the limited time clinicians have to complete cognitive assessments. Lastly, given the low level of emotions triggered by the scenarios, 3DJT is an acceptable test to administer to a more vulnerable clientele, such as seniors.

Regarding the limitations of the present study, the sample size of Phase 2 means that more studies will be required to corroborate our current results. Furthermore, although a significant effort was made to obtain a sample that was as representative as possible of the Quebec population, we obtained an overrepresentation of women, young people, and participants with a high level of education. It should be noted, however, that no correlation could be established between the 3DJT score and the level of education. For logistical reasons, the order of the assessment sessions was reversed for some participants, so some subjects were randomized even though they had to be excluded from Phase 2. It should be noted, however, that the groups had a similar composition despite this. Finally, and in line with the approach we have taken, we want to make some changes to the scoring scale and develop a scoring guide that better facilitates the administration of the test and that takes into account the reality observed in the field (grounded theory).

To conclude, it should be noted that despite the absence of a clinical population in the current study, a phase of patient testing is already planned. This last step will be required to confirm if the 3DJT can be a reliable and valid tool for measuring judgment.

## 5. Conclusions

The 3DJT is a computerized tool aimed at assessing the three major domains of judgment and is intended to be the result of a collaborative co-construction with experts from various disciplines, subjects without cognitive impairment, and eventually patients and their relatives. Such an effort has allowed and will allow the test to be adapted to the needs of end-users, to integrate various perspectives enriching the final product, and to create a cognitive assessment tool based on the reality in the field. So far, study results of the 3DJT’s psychometric properties are promising and would allow for the development of a 6- or 12-item version that could be used in clinical practice. However, more studies will be needed to corroborate the hypothesis that this instrument is a reliable and valid tool for measuring judgment. Therefore, we are planning phase 3, in which we will pursue the validation of the test through a contrasting group study with individuals. This study will include subjects with cognitive disorders in order to confirm if the 3DJT can effectively identify a deterioration in judgment among a population that is at higher risk of having an impairment of this cognitive skill. Finally, we expect to continue our co-construction approach by consulting with patients and other stakeholders in order to continue to improve the quality of 3DJT.

## Figures and Tables

**Table 2:** Intuitive and deliberate processes involved in judgment.

	Deliberate Process	Intuitive Process
Speed	Slow	Fast, almost instantaneous
Control	Voluntary and controllable	Automatic and unconscious
Accessibility	Accessible	Not accessible, except for their results
Attentional resources	Demanding	Do not demand attentional resources (cognitive shortcuts)
Situations	New or complex (e.g., whether to take a loved one off life support after an accident with irreversible neurological damage)	Simple and familiar or trained (e.g., getting up in the middle of the night to get a glass of water because you are thirsty)

**Table 3:** Measures of practical judgment that can be used in the clinic.

	ILS-PS	JAT	KPT	NAB-JDG	NCSE-JQ	TOP-J
Description	33 items 20 to 25 min to complete	7 items (3 for generation of solutions, 4 for assessment of options)	3 situations illustrated with the help of images 5 min to complete	10 items	4 scenarios	15 items 10 min to complete
Psychometric properties	Alpha not published Content validity issue Ceiling effect	Alpha: 0.71–0.85 Kappa: 0.92–0.93 Validation with patients with MA	Alpha: 0.88–0.93, overestimation? Good content validity	Alpha: 0.45 Content validity issue Ceiling effect	Alpha: 0.04–0.46 Content validity issue Ceiling effect	Alpha: 0.63 overestimation? Validation primer with subjects with cognitive complaints, minor neurocognitive disorder, and AD
Comments	Part of a larger assessment Does not detect dementia	Allows for separate evaluation of solution generation and assessment Good concurrent validity Validated in French	Studies within patients with major neurocognitive disorder. Limited assessment of judgment (scenario taking place in a kitchen)	Part of a larger battery of tests Does not detect dementia	Part of a larger battery of tests Deficient guidelines Little normative data to support the interpretation Does not detect dementia	Unidimensionality of judgment

ILS-PS—Problem Solving Subtest of the Independent Living Scale (ILS-PS); JAT—Judgment Assessment Tool; KPT—Kitchen Picture Test; NAB-JDG—Judgment/Daily Living Test of the Neuropsychological Assessment Battery; NCSE-JQ—Neurobehavioral Cognitive Status Exam; TOP-J—Test of Practical Judgment.

**Table 4:** Presentation of three different items from the version used in Phase 2 of the study. Each of the three scenarios assesses one of the three domains of judgment (practical, moral, or social). Of these items, two are Type G (generation of solutions) and one is Type E (evaluation of consequences).

Type of Item	Scenario and Questions
Practical judgment, type G (generation of solutions)	You are taking medication for pressure and to prevent epilepsy. This morning, you mistakenly took two tablets of your epilepsy medication and forgot to take the pressure medication. You realize this half an hour after making the mistake. However, you have no symptoms or discomfort. Type D question: Would you go read on the internet what to do in such a situation? YES or NO. You have 8 s to answer. Type J question: Why? Explain your answer as much as possible. You have one minute to answer. Type G question: Would there be other possible solutions in the context? You have one minute to provide as many solutions as possible.
Moral judgment, type E (evaluation of consequences)	You work at a supermarket. It is a temporary job because next month you start a new job. You notice that one of your colleagues is taking goods belonging to the supermarket home without paying for them. You know that he has a difficult financial situation and a family to support, but an inventory of the products will be carried out next month, and the bosses will look for those responsible for the loss of merchandise. Type D question: Do you report the situation to your boss? YES or NO. You have 8 s to answer. Type J question: Why? Explain your answer as much as possible. You have one minute to answer. Type E question: Name the pros and cons of reporting the situation to your boss. You have one minute to provide as many answers as possible.
Social judgment, type G (generation of solutions)	You have a problem with a leaky sink. You decide to fix it yourself, but first, ask your neighbor for advice. He used to be a janitor in a building but has been retired for a few years. He offers to repair it himself, which you accept. He works very hard for almost an hour and manages to fix the problem. You want to show appreciation for his service. Type D question: Do you offer him an amount of money? YES or NO. You have 8 s to answer. Type J question: Why? Explain your answer as much as possible. You have one minute to answer. Type G question: In what other ways could you show your appreciation for his service? You have one minute to provide as many solutions as possible.

**Table 5:** Criteria used to classify the scenarios according to the results of the experts’ assessments of their content validity, relevance, and acceptability for potential administration to patients.

Accepted	The accepted scenarios had to meet the following criteria:	
	Content validity	Have been classified by six out of eight experts as being in the same area (domain) of judgment.
	Relevance	Have obtained an average score of ≥3/5 for relevance.
	Acceptability	Have been classified by at least six out of eight experts as acceptable or acceptable with minor adjustments.
Eliminated	The eliminated scenarios had to meet one of the following criteria:	
	Content validity	Not having been classified by at least five out of eight experts as being in the same area (domain) of judgment.
	Relevance	Have obtained an average score of <2/5 for relevance.
	Acceptability	Have been classified by at least three out of eight experts as unacceptable for potential administration to patients.
Borderline	Scenarios that meet neither the criteria for “accepted” nor the criteria for “eliminated”	

**Table 6:** Summary of expert comments and suggestions from Phase 1 that were incorporated into the improved version of the 3DJT used for Phase 2.

For the whole test	Make a 6–12 scenario version for clinical use. A longer version could be developed for research; The fact that the 3DJT is a test that assesses the integrity of the processes underlying judgment rather than the decision made by the participant was valued by the experts because it is part of an approach that recognizes pluralism within society (there is not necessarily one correct solution to a situation); Consider cultural and generational differences. The fact that the 3DJT is a tool that focuses more on the assessment of the processes underlying the judgment than the decisions made by the participants provides an opening in this sense.
Format of the test	Improve the audio used to read the statements; In general, pictures are a good complement to illustrate the scenarios.
Instructions	Reduce the length of instructions given to participants; Encourage practice scenarios to ensure that instructions are understood.
Scenarios	Reduce the length of the statements; Show the scenario statement as you present the various questions to the participant so that they have all the information available to answer; Increase the allocated time to answer type D questions. In the enhanced version of the 3DJT, we increased it to 8 s; Type A scenarios could be adapted to measure individuals’ abilities to assess the advantages and disadvantages of solutions. Thus, in the enhanced version of the 3DJT, the type A scenarios were replaced with type E (evaluation of consequences) scenarios. This was carried out to provide more structure to the evaluation conducted using the 3DJT and to facilitate measurements. The experts felt that a more structured assessment might be more appropriate for a neurocognitive clientele; While good ecological validity of the scenarios is generally desirable, it was noted that it may still be interesting to have some scenarios that are less likely to occur in everyday life but raise important issues (e.g., important ethical or social issues); It would be desirable to have scenarios containing some variation in difficulty levels, especially to avoid a ceiling effect.

**Table 7:** Sociodemographic and clinical characteristics of the sample (M ± ST).

Measures	Group A (*n* = 28)	Group B (*n* = 34)	*p*-Value
Age (years)	39.00 ± 18.962	40.74 ± 18.628	0.719
Number of years of education (years)	16.607 ± 2.750	16.956 ± 2.740	0.621
Females, *n* (%)	17 (61%)	20 (59%)	0.880 (X^2^ = 0.023)
Ethnicity	Caucasian: 17	Caucasian: 19	0.701 ^a^ (X^2^ = 0.147)
	Arabic: 5	Arabic: 8	
	Asian: 3	Hispanic: 2	
	Hispanic: 0	Asian: 1	
	Indigenous: 0	Indigenous: 0	
	Oceanic: 0	Oceanic: 0	
	Other: 3	Other: 3	
Marital status	Single: 15	Single: 19	0.856 ^b^ (X^2^ = 0.033)
	Married: 6	Married: 7	
	Common law partner: 5	Common law partner: 5	
	Widow: 2	Divorced or separated: 2	
	Civil union: 0	Widow: 1	
	Divorced or separated: 0	Civil union: 0	
MOCA score	28.04 ± 1.934	27.59 ± 2.076	0.384
MCSDS-13 score	7.82 ± 3.163	7.68 ± 2.682	0.848

M—mean; ST—standard deviation; MOCA—Montreal Cognitive Assessment; MCSDS-13—Marlowe–Crowne Social Desirability Scale 13 items version as developed by Reynolds. ^a^: For analysis, we compared Caucasian versus non-Caucasian. ^b^: For analysis, we compared single versus non-single.

## Data Availability

The datasets used and/or analyzed for the study are available from the corresponding authors upon reasonable request.

## Supplementary Materials

The following supporting information can be downloaded at: https://www.mdpi.com/article/10.3390/jcm12113740/s1, Figure S1: Rating scales used by experts to assess various aspects of each of the vignettes in the 3DJT; Table S1: Composition of the expert panel by gender, profession, and years in practice; Table S2: Results of expert ratings; Table S3: Scenario quality measurements results (healthy participants assessment); Table S4: Results of the correlations between the 3DJT score per scenario and the scores on different cognitive tests; Table S5: Cronbach coefficient of 3DJT subtest.
