# Supplementary material for: Clinical Assessment of Judgment in Adults and the Elderly: Development and Validation of the Three Domains of Judgment Test—Clinical Version (3DJT-CV)
Source: J Clin Med. 2023 May 29;12(11):3740. doi: 10.3390/jcm12113740 (PMC10253541; doi:10.3390/jcm12113740)
Supplement: Supplementary file 1 [file jcm-12-03740-s001.zip › jcm-2316242-supplementary.pdf]

<u>Supplementary Materials</u>

Acceptability scale for administration to patients:

Inacceptable	*Acceptable with major modifications*	*Acceptable with minor modifications*	*Acceptable*

Rating scale to assess the relevance of the item, the clarity of its statement and the relevance and quality of the scenario's images

0	1	2	3	4	5
Very unsatisfied	Unsatisfied	Borderline	Good	Very good	Excellent

Scale to assess the ecological validity of the scenario (Do you think such a situation might occur in a person's daily life?):

0	1	2	3	4	5
Impossible	Very unlikely	Unlikely	Probable	Very probable	Assuringly

Scale to assess scenario difficulty:

0	1	2	3	4	5
Too easy	Very easy	Easy	Moderate	Difficult	Very difficult

Figure S1: Rating scales used by experts to assess various aspects of each of the vignettes in the 3DJT

Table S1. Composition of the expert panel by gender, profession, and years in practice

Gender	Female	25
	Male	7
Profession	Neurology, neuropsychiatry, or consultation-liaison psychiatry	5
	Geriatric psychiatry	10
	Geriatrics	3
	Neuropsychologist	11
	Occupational therapist	3
Years in practice	< 5 years	4
	5-10 years	6
	11-15 years	10
	> 15 years	12

Table S2. Results of expert ratings

The test originally consisted of a total of 72 scenarios divided into three subtests of 24 scenarios, with each subtest assessing one of the three main domains of judgment (moral, practical, and social). Each subtest consisted of 12 Type G scenarios and 12 type A scenarios. After expert evaluation, 18 scenarios assessing practical judgment (10 type G and 8 type A), 18 scenarios assessing moral judgment (8 type G and 10 type A), and 20 scenarios assessing social judgment (11 type G and 9 type A) were accepted for phase 2 of the study.

	**Practical Judgment Subtest**	**Moral Judgment Subtest**	**Social Judgment Subtest**	**Total**
**Type G scenario** (generation of solutions)	10 accepted<br>1 borderline<br>1 eliminated	8 accepted<br>2 borderline<br>2 eliminated	11 accepted<br>1 borderline<br>0 eliminated	29 accepted<br>4 borderline<br>3 eliminated
**Type A scenario** (alternative scenario)	8 accepted<br>2 borderline<br>2 eliminated	10 accepted<br>1 borderline<br>1 eliminated	9 accepted<br>3 borderline<br>0 eliminated	27 accepted<br>6 borderline<br>3 eliminated
**Total**	18 accepted<br>3 borderline<br>3 eliminated	18 accepted<br>3 borderline<br>3 eliminated	20 accepted<br>4 borderline<br>0 eliminated	**56 accepted**<br>**10 borderline**<br>**6 eliminated**

Table S3: Scenario quality measurements results (healthy participants assessment) (M-ÉT)

Results of measures of clarity, ecological validity, difficulty, and level of emotions generated per scenario. Ratings range from 0 to 5, where 0 is a null value and 5 is the maximum value that can be assigned by the participant. M: mean, SD: standard deviation.

Scenario (version-n°)	Clarity	Ecological validity	Difficulty	Emotions triggered
A-1	4.07 ± 0.979	4.07 ± 0.900	2.64 ± 1.129	3.11 ± 1.066
A-2	4.07 ± 0.858	3.64 ± 1.096	2.21 ± 1.067	1.18 ± 1.056
A-3	4.46 ± 0.744	4.11 ± 1.166	1.71 ± 0.763	1.18 ± 1.249
A-4	4.25 ± 0.844	4.04 ± 0.922	1.89 ± 0.944	1.25 ± 1.175
A-5	3.93 ± 0.766	3.93 ± 0.940	2.11 ± 1.133	1.75 ± 1.378
A-6	3.82 ± 0.83	4.32 ± 0.863	2.57 ± 1.168	1.82 ± 1.307
A-7	4.29 ± 0.763	4.07 ± 0.900	1.71 ± 0.854	1.14 ± 1.113
A-8	4.18 ± 0.863	4.14 ± 0.970	1.61 ± 0.737	1.14 ± 1.113
A-9	3.68 ± 0.945	3.32 ± 1.219	2.18 ± 0.983	1.93 ± 1.245
A-10	3.79 ± 0.957	3.93 ± 1.120	2.18 ± 1.124	1.71 ± 1.213
A-11	4.29 ± 0.713	2.68 ± 1.362	2.18 ± 1.124	1.71 ± 1.301
A-12	3.93 ± 0.940	4.29 ± 0.854	2.00 ± 0.943	1.14 ± 1.177
A-13	4.14 ± 0.848	4.25 ± 0.844	1.79 ± 0.876	1.14 ± 1.145
A-14	3.71 ± 1.013	4.18 ± 1.056	2.07 ± 0.979	1.18 ± 1.188
A-15	4.21 ± 0.876	3.71 ± 0.897	2.04 ± 1.036	2.54 ± 1.319
A-16	3.96 ± 1.036	3.57 ± 1.103	2.07 ± 0.979	2.46 ± 1.319
A-17	4.11 ± 0.875	3.43 ± 0.959	1.71 ± 1.013	1.11 ± 0.994
A-18	4.18 ± 0.772	3.00 ± 1.388	2.18 ± 1.124	2.61 ± 1.370
A-19	4.11 ± 0.875	3.64 ± 1.096	1.75 ± 0.799	1.46 ± 1.105
A-20	4.00 ± 1.018	3.21 ± 1.343	2.75 ± 1.206	2.57 ± 1.317
A-21	4.07 ± 0.940	2.96 ± 1.201	2.32 ± 1.020	1.39 ± 1.257
A-22	4.07 ± 0.900	2.82 ± 1.335	2.04 ± 1.177	1.93 ± 1.274
A-23	4.32 ± 0.670	3.11 ± 1.286	2.07 ± 1.331	2.00 ± 1.540
A-24	4.15 ± 0.818	2.93 ± 1.141	2.67 ± 1.177	2.70 ± 1.382
A-25	4.21 ± 0.787	2.93 ± 1.359	2.11 ± 1.031	1.50 ± 1.262
A-26	4.29 ± 0.854	3.11 ± 1.423	2.25 ± 1.076	1.61 ± 1.315
A-27	3.82 ± 0.905	4.00 ± 1.388	2.04 ± 1.170	0.71 ± 0.976
A-28	4.07 ± 0.900	4.25 ± 1.005	1.93 ± 0.900	1.32 ± 1.219
B-1	4.47± 0.788	4.32 ± 0.945	2.03 ± 0.969	1.24 ± 1.075
B-2	4.50 ± 0.615	1.59 ± 1.158	2.03 ± 0.904	1.76 ± 1.130
B-3	4.32 ± 0.727	3.88 ± 0.844	1.94 ± 0.776	1.56 ± 1.160
B-4	4.47 ± 0.615	4.50 ± 0.749	1.50 ± 0.615	2.09 ± 1.334
B-5	4.21 ± 0.845	3.06 ± 1.229	1.88 ± 0.913	1.62 ± 1.155
B-6	4.18 ± 0.869	3.18 ± 1.267	1.94 ± 0.983	1.26 ± 1.263
B-7	4.38 ± 0.817	4.12 ± 0.769	1.97 ± 0.904	1.74 ± 1.310
B-8	4.26 ± 0.751	2.91 ± 1.083	2.09 ± 1.111	2.12 ± 1.452
B-9	4.44 ± 0.746	4.41 ± 0.857	1.82 ± 1.058	2.24 ± 1.458
B-10	4.38 ± 0.779	4.26 ± 1.286	1.47 ± 0.961	1.32 ± 1.093
B-11	4.29 ± 0.760	3.21 ± 1.067	2.32 ± 1.224	2.65 ± 1.203
B-12	4.26 ± 0.790	3.82 ± 0.936	2.03 ± 0.937	1.97 ± 1.314
B-13	4.44 ± 0.660	3.91 ± 1.264	1.65 ± 0.849	1.26 ± 1.214

B-14	4.29 ± 0.836	2.59 ± 1.019	2.24 ± 1.046	2.35 ± 1.323
B-15	4.32 ± 0.843	3.29 ± 1.244	1.85 ± 0.857	1.44 ± 1.307
B-16	4.21 ± 0.770	3.97 ± 1.058	2.21 ± 0.978	1.85 ± 1.234
B-17	4.47 ± 0.706	3.85 ± 1.048	1.62 ± 0.652	1.76 ± 1.394
B-18	4.24 ± 0.741	2.82 ± 0.968	2.03 ± 0.870	1.71 ± 1.382
B-19	4.32 ± 0.684	3.71 ± 1.031	1.76 ± 0.781	1.21 ± 1.067
B-20	4.32 ± 0.684	3.94 ± 1.153	1.65 ± 0.774	1.47 ± 1.080
B-21	4.32 ± 0.684	4.03 ± 1.000	1.88 ± 0.977	1.68 ± 1.364
B-22	4.32 ± 0.727	3.12 ± 1.066	2.74 ± 1.163	2.50 ± 1.441
B-23	4.24 ± 0.781	3.56 ± 1.236	2.12 ± 0.844	1.74 ± 1.163
B-24	4.00 ± 0.921	3.56 ± 1.353	1.82 ± 0.834	1.21 ± 1.067
B-25	4.21 ± 0.770	2.53 ± 0.961	2.62 ± 1.280	2.56 ± 1.397
B-26	4.18 ± 0.869	3.21 ± 1.493	1.94 ± 0.983	1.32 ± 1.296
B-27	4.00 ± 1.015	2.03 ± 1.243	3.44 ± 1.418	2.88 ± 1.409
B-28	4.41 ± 0.657	4.53 ± 0.706	1.56 ± 0.660	0.88 ± 0.880

Table S4 : Results of correlations between the 3DJT score per scenario and the scores on different cognitive tests

The scenarios whose designation starts with the letter A correspond to version A and the ones whose designation starts with the letter B to version B.

Scenarios	Type	JAT	Rey	Fluency	Emotions
A-1	M type E	0.432*	-0.278	0.450*	-0.031
A-2	P type G	0.397*	0.043	0.222	0.058
A-3	S type E	0.147	-0.149	0.272	0.416*
A-4	S type G	0.176	-0.464*	0.106	-0.089
A-5	M type G	0.339†	-0.230	0.269	0.168
A-6	P type E	-0.007	-0.076	0.092	0.101
A-7	S type G	0.299	-0.181	0.191	0.079
A-8	P type G	0.156	-0.334†	0.326†	0.015
A-9	P type E	0.362†	-0.122	0.221	0.102
A-10	M type G	0.425*	-0.230	0.479**	-0.069
A-11	M type E	0.330†	-0.330†	0.307	0.076
A-12	S type E	0.526**	-0.484*	0.325†	0.040
A-13	P type G	0.230	-0.410*	0.288	-0.075
A-14	P type G	-0.039	-0.329†	-0.066	-0.241
A-15	M type E	0.341†	-0.236	0.451*	0.344†
A-16	S type G	0.163	-0.097	0.247	0.332†
A-17	P type G	0.048	-0.003	0.271	0.012
A-18	M type G	0.207	-0.167	0.351†	0.055
A-19	S type G	0.113	-0.089	0.107	0.159
A-20	M type E	0.403*	-0.004	0.229	0.212
A-21	S type G	0.361†	0.253	0.201	-0.084
A-22	M type G	0.292	-0.019	0.346†	0.242
A-23	P type E	0.376*	-0.038	0.428*	0.242
A-24	M type E	0.448*	-0.107	0.458*	0.265
A-25	S type E	0.443*	-0.117	0.386*	0.388*

A-26	S type G	0.358†	-0.182	0.359†	0.318†
A-27	P type E	0.403*	-0.001	0.414*	0.393*
A-28	S type E	0.566**	-0.130	0.392*	0.350†
B-1	S type E	0.364*	-0.326†	0.472**	-0.087
B-2	M type G	0.263	-0.059	0.218	0.326†
B-3	P type G	0.372*	-0.297	0.343*	0.048
B-4	S type G	0.258	-0.102	0.163	0.021
B-5	S type E	0.161	-0.265	0.382*	0.077
B-6	M type E	0.462**	-0.255	0.188	0.197
B-7	P type E	0.276	-0.139	0.356*	0.001
B-8	M type E	0.372*	-0.139	0.306†	0.038
B-9	S type E	0.196	0.015	0.177	0.090
B-10	P type G	0.423*	-0.271	0.433*	0.071
B-11	M type E	0.216	0.078	-0.089	-0.054
B-12	S type G	0.069	-0.052	0.285	0.084
B-13	S type E	0.261	0.054	0.264	0.031
B-14	P type E	0.529**	-0.119	0.313†	0.146
B-15	P type G	0.136	-0.152	0.236	0.108
B-16	M type E	0.239	-0.143	0.075	0.030
B-17	S type G	0.554**	-0.131	0.443**	0.177
B-18	M type G	0.371*	-0.122	0.546**	0.093
B-19	S type G	0.392*	-0.176	0.367*	-0.047
B-20	S type G	0.374*	-0.014	0.304†	0.125
B-21	P type E	0.152	-0.269	0.430*	-0.022
B-22	M type G	0.233	-0.223	0.367*	0.086
B-23	S type E	0.425*	-0.235	0.518**	0.117
B-24	P type G	0.331†	-0.002	0.404*	0.034
B-25	M type G	-0.099	-0.090	0.197	0.031
B-26	P type E	0.427*	0.107	0.342*	0.194

B-27	M type E	0.094	-0.263	0.351*	0.103
B-28	P type G	0.241	0.010	0.512**	0.096

P: scenarios of the practical judgment assessment subtest, M: scenarios of the moral judgment assessment subtest, S: scenarios of the social judgment assessment subtest, G: scenario measuring solution generation, E: scenario measuring solution evaluation, JAT: Judgment Assessment Tool, Rey: 15-word Test (number of trials needed to correctly recall the 15-word list), Fluency: Semantic Fluency Test, Emotions: Facial Emotions Stimuli (Ekman and Friesen), *: significant results ($p < 0.05$), **: highly significant results ($p \leq 0.01$), †: statistical trend ($0.10 \geq p$ value > 0.05).

Table S5: Cronbach coefficient

Cronbach's alphas are presented by subtest (practical, moral or social) and by version of the test (A or B).

**Version and subtest**	**Cronbach alpha**
Version A – Practical judgment scenarios	0.830
Version B – Practical judgment scenarios	0.841
Version A – Moral judgment scenarios	0.883
Version B – Moral judgment scenarios	0.776
Version A – Social judgment scenarios	0.858
Version B – Social judgment scenarios	0.876
